# PGPRs and nitrogen-fixing legumes: a perfect team for efficient Cd phytoremediation?

**DOI:** 10.3389/fpls.2015.00081

**Published:** 2015-02-25

**Authors:** María T. Gómez-Sagasti, Daniel Marino

**Affiliations:** ^1^Laboratory of Plant Physiology, Department of Plant Biology and Ecology, University of the Basque CountryBilbao, Spain; ^2^Ikerbasque, Basque Foundation for ScienceBilbao, Spain

**Keywords:** cadmium, PGPRs, legume, nitrogen fixation, symbiosis, phytoremediation

## Abstract

Cadmium (Cd) is a toxic, biologically non-essential and highly mobile metal that has become an increasingly important environmental hazard to both wildlife and humans. In contrast to conventional remediation technologies, phytoremediation based on legume–rhizobia symbiosis has emerged as an inexpensive decontamination alternative which also revitalize contaminated soils due to the role of legumes in nitrogen cycling. In recent years, there is a growing interest in understanding symbiotic legume–rhizobia relationship and its interactions with Cd. The aim of the present review is to provide a comprehensive picture of the main effects of Cd in N_2_-fixing leguminous plants and the benefits of exploiting this symbiosis together with plant growth promoting rhizobacteria to boost an efficient reclamation of Cd-contaminated soils.

## LEGUMES, A PROMISING TOOL FOR CD PHYTOREMEDIATION

An increasingly industrialized global economy over the last century has led to a dramatic increase in production and release of hazardous metals to the environment (Gerhardt et al., 2009). Among all the non-essential metals, cadmium (Cd) has received great attention in soil science and plant nutrition mainly due to (1) its phytotoxic impact ranging from growth reduction, wilting, and chlorosis to cell death (Gallego et al., 2012); (2) its relative high mobility in the soil–plant system, which implies Cd dissemination throughout the food chain, even becoming a serious threat to ecosystem and human health (Burger, 2008) and; (3) its long half-life in soil system varying between 100 and 1,000 years (Central Pollution Control Board [CPCB], 2007). The resulting detrimental effects derived from excess of Cd on environment and human health are well documented (Clemens et al., 2013).

Today, environmental managers are increasingly becoming aware of the importance to remediate Cd-contaminated areas using biological systems (microorganisms and/or plants), which are more ecologically sound, less labor-intensive, safe, and economically advantageous than conventional methods based on physico-chemical processes (e.g., land filling, chemical fixation, and leaching). Concerning bioremediation, plant-assisted remediation or phytoremediation has been highlighted for its potential for *in situ* removal of Cd from soils (Salt et al., 1995). Phytoremediation of Cd-contaminated soils encompasses three different strategies: (1) phytoextraction (uptake and accumulation of metal from soils into the plant’s harvestable parts); (2) phytostabilization (complexation of metal in the rhizosediment decreasing its solubility/bioavailability) and; (3) rhizofiltration (absorption of metal by roots).

Although phytoremediation is a promising technology, its feasibility depends on site conditions, soil properties, and plants sensitivity to the toxic metal. In particular, the poor soil structure, low water-holding capacity, lack of organic matter (OM) and its associated nutrients such as nitrogen (N) and phosphorous (P) are some of the distinctive features of metal-polluted soils that are a matter of importance in the early stages of phyremediation (i.e., in the establishment of plant cover; Wong, 2003). In this regard, the exploitation of symbiotic relationship between leguminous plants and rhizobia is presented as an attractive and cost-effective alternative to improve the nitrogen input into the plant–soil system compared with the use of expensive synthetic N-fertilizers (United States Department of Agriculture, Economic Research Service [USDA-ERS], 2013). Moreover, the contribution of soil bacteria, other than rhizobia, to improve the metal remediation capacity of symbiotic legumes represents a growing area of research. These bacteria are known as plant growth-promoting rhizobacteria (PGPRs). This review summarizes some of the recent advances in this field and highlights the potential of this three partner relationship legume–rhizobia–PGPRs for Cd detoxification.

Legumes (*Fabaceae* or *Leguminosae*) is the third largest angiosperm family, with more than 700 genera and 18,000 species with an exceptionally wide range of habitats (Lewis et al., 2005). One of the outstanding characteristics of this family is that most legumes have the ability to establish a symbiotic relationship with soil nitrogen-fixing (N_2_-fixing) rhizobacteria, known collectively as rhizobia, e.g., *Rhizobium*, *Mesorhizobium*, *Bradyrhizobium*, *Azorhizobium*, *Allorhizobium,* or *Sinorhizobium* (Velázquez et al., 2010).

The symbiotic process is initiated with the production of Nod Factors (NFs) by rhizobia in response to plant root exudates containing (iso)flavonoids. The perception of NFs by the plant will then launch the bacterial infection (Oldroyd, 2013). This molecular dialog culminates in the formation of a new organ, the nodules, which are formed in the roots and in rare cases in the stems. Inside the nodules, the symbiotic nitrogen fixation (SNF) process takes places. Plants provide a carbon source to the bacteria to fuel the energy demand of the SNF and also a microaerophilic environment inside the nodules, which is compatible with nitrogenase (Nase) complex functioning. The enzyme Nase reduces the atmospheric dinitrogen to ammonia, which will be incorporated into organic forms and then exported from the nodules to sustain plant growth (Oldroyd, 2013).

Thus, SNF also makes legumes ideal pioneers to colonize and restore the quality and health of N-limited environments, a common feature of metal-contaminated soils (Zaidi et al., 2012). This capacity together with legumes deep-reaching root system and high biomass are ideal traits for efficient phytoremediation of Cd.

## LEGUME–RHIZOBIA SYMBIOSIS IS SENSITIVE TO CD

Cadmium is a very toxic element even at low concentrations, being ranked number 7 by the Agency for Toxic Substances and Disease Registry (ATSDR, 2013). The exposure to Cd can affect considerably the symbiosis establishment, nodule formation and SNF. Cd inhibitory effects on nodulation and SNF depend on the Cd concentration, its bioavailability in the plant growth conditions (agar plates, hydroponics, soil, etc.), the length of the exposure (gradual exposure to low concentrations or a severe shock), and the specific sensibility of species. Main Cd effects on legume nodules are summarized in **Table 1**.

**Table 1:** Summary of the main deleterious cadmium effects reported in legume nodules.

Cadmium effects*	Legume
**Nodule formation and functioning**
Nodule number/weight	*Arachis hypogaea* (Bianucci et al., 2013); *Cajanus cajan* (Garg and Aggarwal, 2011); *Glycine max* (Chen et al., 2003); *Lupinus albus* (Carpena et al., 2003); *Medicago sativa* (Neumann et al., 1998); *Pisum sativum* (Hernández et al., 1995); *Vigna radiata* (Muneer et al., 2012)
Nodule ultrastructure alteration	*A. hypogaea* (Bianucci et al., 2013); *G. max* (Chen et al., 2003); *L. albus* (Carpena et al., 2003); *M. sativa* (Shvaleva et al., 2010)
Symbiotic nitrogen fixation (SNF) inhibition	*C. cajan* (Garg and Aggarwal, 2011); *G. max* (Balestrasse et al., 2004); *L. albus* (Sánchez-Pardo et al., 2013); *M. sativa* (Shvaleva et al., 2010); *Medicago truncatula* (Marino et al., 2013); *P. sativum* (Hernández et al., 1995)
**Oxygen control**
Reactive oxygen species (ROS) over-production	*A. hypogaea* (Bianucci et al., 2013); *C. cajan* (Garg and Bhandari, 2012)
Oxidative damage	*A. hypogaea* (Bianucci et al., 2013); *C. cajan* (Garg and Aggarwal, 2011); *G. max* (Balestrasse et al., 2004); *L. albus* (Sánchez-Pardo et al., 2013); *Phaseolus vulgaris* (Loscos et al., 2008)
Antioxidant system deregulation	*C. cajan* (Garg and Aggarwal, 2011); *G. max* (Balestrasse et al., 2004); *L. albus* (Carpena et al., 2003); *M. sativa* (Shvaleva et al., 2010); *M. truncatula* (Marino et al., 2013); *P. vulgaris* (Loscos et al., 2008); *V. radiata* (Muneer et al., 2012)
Leghemoglobin (Lb) degradation	*C. cajan* (Garg and Aggarwal, 2011); *G. max* (Balestrasse et al., 2004); *L. albus* (Carpena et al., 2003); *M. truncatula* (Marino et al., 2013); *P. vulgaris* (Loscos et al., 2008); *V. radiata* (Muneer et al., 2012)
**Primary metabolism**
Nitrogen assimilation	*G. max* (Balestrasse et al., 2006); *L. albus* (Sánchez-Pardo et al., 2013)
Carbon metabolism/balance alteration	*L. albus* (Sánchez-Pardo et al., 2013)

*These effects are dependent on Cd concentration, exposure time, plant species, and growth conditions.

The harmful outcome of Cd on nitrogen fixation is in part due to a direct effect of Cd in the survival of free-living rhizobia in the soil (Smith, 1997; Giller et al., 1998), which results even in their gradual extinction (Broos et al., 2005). For instance, effective *Rhizobium leguminosarum* bv. *trifolii* population did not survive after long-term incubation of soils containing 7.1 mg Cd kg^-1^ (Chaudri et al., 1992) and soils amended with metal-enriched liquid sludge and metal salts began to show impacts on rhizobia over time (11 year time-lapse; Chaudri et al., 2008).

Besides the potential deleterious effects of Cd on the growth and survival of rhizobia, nodulation, and the morphology of the nodules are also considerably affected. For instance, the addition of 16 and 20 mg Cd kg^-1^ soil caused great inhibition of root growth and nodulation in soybean (*Glycine max*; Chen et al., 2003; Sheirdil et al., 2012). Manier et al. (2009) conducted a specialized “rhizotron” experiment exposing white clover (*Trifolium repens*) to fourteen topsoils from a strongly metal-contaminated (Cd, Zn, and Pb) area and observed a significant decrease in nodulation index (i.e., the number of nodules per gram of the total fresh biomass) at about 2.64 mg Cd kg^-1^ in these soils. The structure of the nodule was also negatively influenced by Cd exposure in white lupin (*Lupinus albus*), resulting in an occluded intracellular spaces of nodule cortex, alterations in symbiosomes, enrichment in Cd of cell walls and, finally, reduction of effective N_2_-fixing area (Carpena et al., 2003).

One of the most known effects related to Cd toxicity in legumes nodules is the overproduction of reactive oxygen species (ROS). In general, the mechanism underlying ROS generation upon Cd exposure remains to be elucidated. Although Cd itself is not redox active since it is not able to trigger the Fenton-type reactions (Salin, 1988), Cd-related ROS production can be indirectly linked to impairment of the antioxidant machinery (Sandalio et al., 2001), to disruption of the electron transport chain, and somehow to the activation of NADPH oxidases in membranes (Romero-Puertas et al., 2004; Cuypers et al., 2010).

Symbiotic nitrogen fixation inhibition related to ROS overproduction in legume nodules has been shown to be related to three different motives; (1) a direct inactivation of Nase, which is extremely sensitive to oxygen and to oxidation by ROS (Naya et al., 2007); (2) leghemoglobin (Lb) degradation, a protein in charge of binding free O_2_ in the infected cells cytosol to supply to the bacteroids for their respiration (Mathieu et al., 1998; Marino et al., 2006); and (3) sucrose synthase down-regulation, a key enzyme in nodule carbon metabolism that hydrolyses the sucrose coming from the photosynthetic process to load the bacteroids with carbon skeletons for energy obtaining (Marino et al., 2009).

When legumes have been exposed to high Cd concentrations or long exposure, the high mobility of Cd brought its translocation to shoots and provoked photosynthesis impairment, leaves chlorosis, and oxidative damage in nodules. So, it seems that the nitrogen fixation inhibition related to a severe Cd exposure observed in different legumes like soybean (Balestrasse et al., 2006), white lupin (Carpena et al., 2003), or mung bean (*Vigna radiata*; Muneer et al., 2012) would be associated to a general plant breakdown rather than to a specific effect of Cd in nodules affecting SNF. However, in a recent work with nodulated *Medicago truncatula* plants grown in split-root system, the differential application of Cd to one part of the root led to a specific activation of nodule antioxidant machinery and a concomitant inhibition of SNF (Marino et al., 2013). In that work, SNF inhibition was related to Lb and Nase down-regulation, whilst sucrose synthase did not vary compared to controls (Marino et al., 2013). This is in agreement with other works showing that Cd application provoked a rapid decrease in Lb content, for instance in soybean (Balestrasse et al., 2004) and common bean (*Phaseolus vulgaris*; Loscos et al., 2008). Interestingly, the heterologous overexpression of a flavodoxin from the filamentous cyanobacterium *Anabaena variabilis* in *Sinorhizobium meliloti* partially prevented Cd toxicity effects on Nase activity in alfalfa (*Medicago sativa*; Shvaleva et al., 2010). Flavodoxins are prokaryotic electron carrier proteins and have been suggested to play a positive role in ROS detoxification (Redondo et al., 2009). In general, among the different regulation pathways that control SNF under abiotic stresses, initial Cd effects on *Medicago* sp. nodules nitrogen fixing capacity seem to be related to Nase down-regulation. This inhibition could be a consequence of the effect reported on Lb, resulting in intracellular free-O_2_ increase that could damage Nase. In contrast, although in other legume species the effect of ROS-producing abiotic stresses has been related to carbon limitation, this does not seem to be the principal inductor of SNF inhibition in *Medicago* sp. (Larrainzar et al., 2014).

**FIGURE 1:**
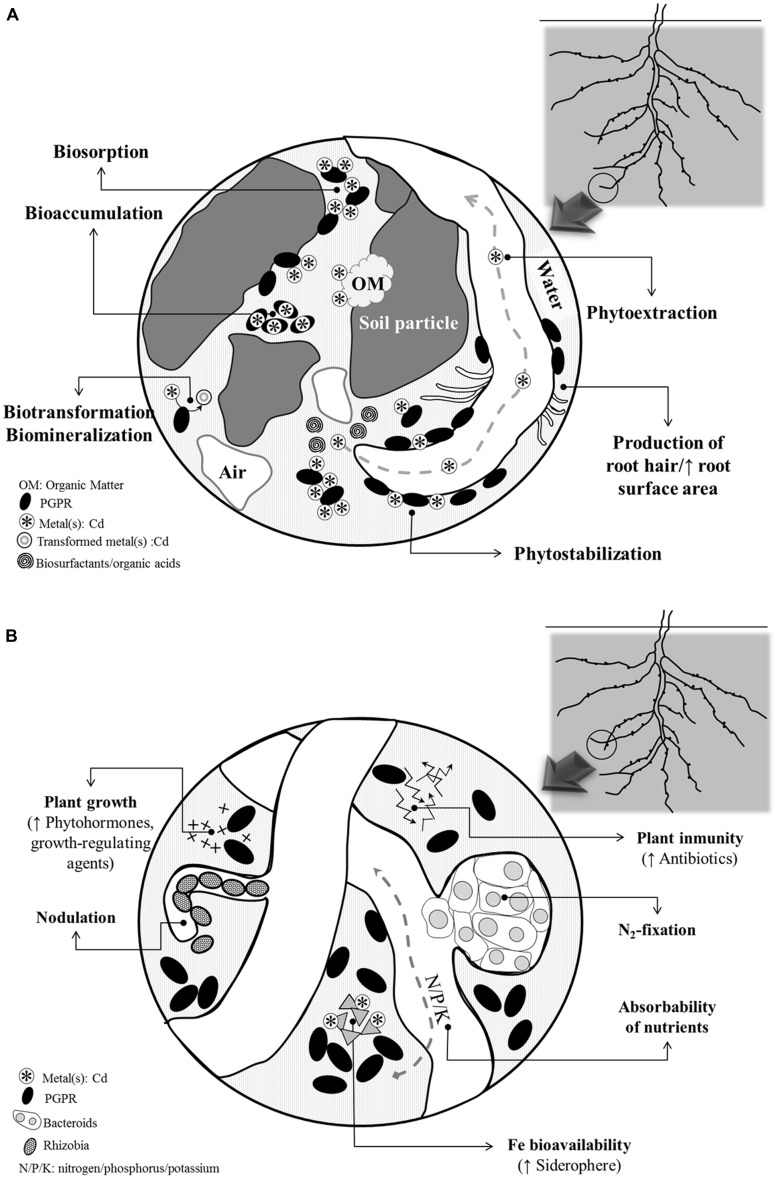
**Summary of the main processes that **(A)** influence metal bio/phytoremediation efficiency during PGPRs-plant interaction and **(B)** are benefited from the interaction between PGPRs and N_**2**_-fixing legumes**.

Since legumes are sensitive to Cd, selecting legume species or genotypes with increased tolerance to Cd is a must to promote their use in remediation of Cd-contaminated soils (Ahmad et al., 2012a). In this perspective, a number of legumes, especially *Anthyllis, Cytisus*, *Lotus*, *Lupinus, Genista*, *Glycine*, *Ononis*, *Ornithopus*, *Pisum*, several *Trifolium* species, *Vicia*, etc (Pajuelo et al., 2007), have shown encouraging results and they have been proposed as promising tools for reclamation of metal-contaminated areas. As occurs with other plant species, the success of legume-based phytoremediation depends mainly on three factors; (1) the metal disponibility for the plant; (2) the capacity of the legumes to cope with metal toxicity; and (3) the ability of legumes for immobilizing Cd in roots (ideal for the phytostabilization purposes) and its uptake, translocation and accumulation in shoots (key features for phytoextraction; Sessitsch et al., 2013). Considering these bottlenecks, in recent years, researchers have taken advantage of rhizosphere inhabitants/rhizobacteria associated with legumes to maximize their capacity/effectiveness to phytoremediate Cd-polluted soils (**Figure 1A**). In this context, PGPRs deserve special attention because of their wide variety of benefits that often enhance plant performance (Mehboob et al., 2013; **Figure 1**).

## THE “HELPER” ROLE OF PGPRs TO LEGUME–RHIZOBIA SYMBIOSIS IN THE LIGHT OF PHYTOREMEDIATION

Broadly, PGPRs may live inside the plant or in the rhizosphere. PGPRs include free-living N_2_-fixing bacteria that prompt plant growth viz., *Azospirillum*, *Azotobacter*, *Acetobacter Bacillus, Burkholderia*, *Azoarcus,* and several species of the family *Enterobacteriaceae* (Hayat et al., 2010). Plant fitness stimulation by PGPRs is achieved by a set of traits which include: synthesis and providing of growth precursors, enhancement of nutrient acquisition, and their beneficial role as biocontrol agents against phytopathogens (reviewed in Lichtfouse, 2009 and Mehboob et al., 2013). More interestingly, PGPRs can interact synergistically, or function as “helper” bacteria to improve the performance of SNF (**Figure 1B**). Basically, PGPRs enhance SNF through promoting root development in general and root hair formation in particular, resulting in more potential colonization sites for rhizobia. In this regard, numerous studies stand out the mechanisms of action of PGPRs (Ahemad and Kibret, 2014). Also, SNF improvement has been associated to a direct effect of PGPRs on nodule metabolism, although how this is achieved is not still known.

Related to root development, PGPRs stimulate SNF through four interrelated traits: (1) the systematic induction of secondary metabolites such as flavonoids in root exudates and B-group vitamins and phytohormones like auxins, citokinins, and gibberellins; (2) the control of low levels of ethylene by the 1-aminocyclopropane-1-carboxylic acid (ACC)-deaminase. This enzyme catalyzes the conversion of ACC, the immediate precursor of ethylene synthesis in plants, to ammonia and α-ketobutyrate; (3) the solubilisation and uptake of soil nutrients (particularly, N, P, and K); and (4) the production and secretion of sideropheres (i.e., low-molecular mass iron chelators that solubilize iron from minerals or organic compounds).

The combined inoculation of *Rhizobium tropici* CIAT899 and *Rhizobium etli* ISP42 together with *Azospirillum brasilense* on common bean, promoted seedlings root branching, and allowed a longer and more persistent exudation of nod-gene-inducing flavonoids that, ultimately, had positive effect on nodule organogenesis (Dardanelli et al., 2008). Moreover, indole acetic acid (IAA) production and ACC-deaminase activity of *Azospirillum* played an important role in common bean nodulation response, particularly under low P conditions in field trial (Remans et al., 2008). In this sense, Cassán et al. (2009) also observed that IAA, gibberellic acid (G3), and zeatin (Z) synthesis was promoted by the cooperative interaction between *A. brasilense* and *Bradyrhizobium japonicum* E109 in soybean. The authors suggested that the over-production of these molecules was behind the enhancement of legumes rhizobial infection, nodule formation, and SNF. Previous work using *Bacillus* sp. also underlined the ability of these bacteria to enhance IAA, G3, and Z content together with nodule Lb concentration, Nase activity, and N_2_-fixation efficiency in common bean (Figueiredo et al., 2008). Additionally, in a field experiment also with common bean, N_2_-fixing *Bacillus subtilis* (OSU-142) and P-solubilizing *Bacillus megaterium* coinoculation with *R. leguminosarum* bv. *phaseoli* increased N and P solubility and, as a result, plants experienced an increase in nodulation and an improvement in growth and yield parameters (Elkoca et al., 2010). Furthermore, some *Bacillus* sp. prompted nodulation and SNF of pea (*Pisum sativum*) by phosphate solubilisation (Mishra et al., 2009a) and IAA production (Mishra et al., 2009b) and enhanced nodulation of pigeon pea (*Cajanus cajan*) by the production and secretion of sideropheres (Rajendran et al., 2008).

In addition to the above mentioned studies, it has been found that pseudomonads in consortium with *Bradyrhizobium japonicum* and *R. phaseoli* caused a significant increase of ACC-deaminase activity resulting in decreased levels of ethylene, which in turn was positively correlated with root elongation and enhanced nodulation in mung bean (Shaharoona et al., 2006; Ahmad et al., 2012b) and lentil plants (*Lens culinaris*; Zahir et al., 2011; Iqbal et al., 2012). Mishra et al. (2011) also demonstrated that the coinoculation of *Pseudomonas* sp. and *R. leguminosarum* increased Lb content (46%) and the total iron (116%) of *L. culinaris* compared to the inoculation with *R. leguminosarum* alone, presumably due to microbial siderophore utilization. Moreover, the authors showed an increase in total N (52%) and P (89%) uptake and suggested that it was the result of root growth stimulation due to IAA production. The importance of IAA production in nodule formation enhancement by *Pseudomonas* strains has also been evidenced in the symbiosis of *Rhizobium galegae-Galega orientalis* (Egamberdieva et al., 2010), *Bradyrhizobium*-mung bean (Malik and Sindhu, 2011) and *Sinorhizobium medicae*-*M. truncatula* (Fox et al., 2011). After *in vitro*, glasshouse, and field experiments, Verma et al. (2012) observed that the dual inoculation of the helper PGPR *Pseudomonas aeruginosa* with *Mesorhizobium* sp. favored the acquisition of P and Fe in chickpea (*Cicer arietinum*) as a consequence of higher production of organic acids and siderophores, respectively. Furthermore, *P. aeruginosa* also increased significantly IAA production in chickpea, which ultimately stimulated root growth and the performance of nodulation and N_2_-fixation compared to inoculation with *Mesorhizobium* sp. alone.

However, little field research has been done to confirm the satisfactory results obtained under controlled experimental conditions (**Table 2**) surely due to the survival disadvantage of inoculated PGPRs in field trials as compared to well-adapted native strains (Rajkumar et al., 2012). Recent advances in molecular biology are contributing to overcome these experimental inconsistencies. Considerable attention has been directed toward genetic engineering of PGPRs and rhizobia to construct significantly improved strains, which express genes that confer adaptive characteristics to site-specific conditions as well as traits associated with plant growth promotion and metal tolerance (Zhuang et al., 2007). Indeed, recombinant PGPRs and rhizobia are advantageous for the expression of foreing genes coming from higher organisms like those encoding metallothioneins including phytochelatins. For instance, a recombinant *Mesorrizobium huakuii* carrying a tetrameric metallothionein (*MTL4*) and a phytochelatin synthase from *Arabidopsis thaliana* (*AtPCS*) favored Cd immobilization in nodules instead of stimulating its translocation in *Astragalus sinicus* (Ike et al., 2007). This is likely to ensure the establishment and survival of introduced PGPRs inoculants at the same time that increase the efficiency of phytoremediation. Nevertheless, regulatory issues and public acceptance of genetically engineered organisms may delay their commercialization and application (Kumar, 2012).

**Table 2:** N_**2**_-fixing legumes and PGPRs assisted phytoremediation of Cd-contaminated soils.

Legume	PGPR	Benefits (under Cd threat)	Potential use	Reference
*Phaseolus vulgaris*	Siderophore-producing bacterial strain KNP9 (probably a strain of *Pseudomonas putida*)	↑ Siderophore production ↑ Root and shoot growth (height and weight) ↑ Chlorophyll content	PS	Tripathi et al. (2005)*
*Pisum sativum*	*Pseudomonas brassicacearum* strain Am3	↑ Cd accumulation in plants ↓ Shoot and seed biomass and P accumulation	PX	Engqvist et al. (2006)
*Pisum sativum*	*P. brassicacearum* Am3, *Pseudomonas marginalis* Dp1, *Rhodococcus* sp. Fp2	↑ Root and shoot biomass ↑ ACC-deaminase activity protecting pea plants from growth inhibition ↑Mineral uptake (N, K, Ca, Fe)	-	Safronova et al. (2006)
*Vigna mungo*	*Pseudomonas aeruginosa* strains (MKRh1, MKRh3 and MKRh4)(rhizosphere/native)	↑ Height, fresh and dry weight of roots and shoots ↑Extensive rooting ↓ Cd accumulation ↑ ACC-deaminase activity, IAA production, siderophore secretion and phosphate solubilization	PS	Ganesan (2008)
*Lupinus luteus*	Heavy metal resistant PGPRs (rhizosphere/native)	↑ Biomass production and N content ↓ Metals accumulation, especially in roots ↓ Metals translocated to the shoots	PS	Dary et al. (2010)*
*Glycine max*	Acidophilic *P. putida* 62BN and alkalophilic *Pseudomonas monteilli* 97AN strains	↓ Cd concentration in plant and soil in their respective soil types	PS	Rani et al. (2009)
*Glycine max*	Cd-tolerant bacteria isolates from nodules of *Glycine max* grown in heavy metal-contaminated soil (rhizosphere/native)	↑ Plant growth via IAA and siderophore production ↑ ACC-deaminase activity and solubilisation of inorganic phosphate ↓ Cd accumulation by increasing Fe (and other mineral nutrients) availability (compared to *Lolium multiflorum*)	PS	Guo and Chi (2014)

- Not mentioned. ↑ Increase; ↓ Decrease; PS, phytostabilization; PX, Phytoextraction. *Cd and other metal(s).

Overall, together with the inherent bacterial characteristic to biosorb metals, PGPRs are benefitial for the symbiotic interaction thanks to the regulation of plant hormone balance, notably by IAA production and through ethylene level control. In addition, Lb control of nodule low O_2_ levels is essential to protect Nase upon Cd exposure and PGPRs have been shown to somehow increase Lb content which could be a key aspect to sustain SNF under Cd stress. Besides, PGPRs-legume interaction also supports the establishment of seedlings and improves the vitality of legumes during metal phytoestabilization and phytoextraction strategies (Shilev et al., 2012). In **Table 2** we have summarized some of the recent studies conducted with the aim of advancing in PGPRs-legumes interaction in the interest of Cd-phytoremediation.

## CONCLUSION AND PERSPECTIVES

Extensive research on the valuable cooperation of PGPRs and N_2_-fixing legumes for phytoremediation purposes has been performed and it is ongoing due to its enormous potential to renew Cd-contaminated soils. However, there are several knowledge barriers which need to be addressed. Prominent among them are optimization of SNF under stressful conditions and a greater understanding of the ecology and dynamics of PGPRs under field conditions. In this respect, before inoculating soils with PGPRs, it must be considered that some strains might be pathogenic to some plant species and even allergenic for humans. Moreover, if the strains inoculated have been genetically modified the potential of horizontal gene transfer should be born in mind. It is also especially important the use and safe disposal of legume edible parts after phytoremediation process (i.e., roots, shoots, and seeds), since they could constitute an important route of Cd introduction in the food chain. For this reason, legumes used as phytoremediation tools should not be considered as products for animal feed or human consumption.

Finally, to boost the use of PGPRs–rhizobia–legume partnership the use of metagenomic approaches are essential to identify new bacterial strains with PGPR traits. Moreover, research should be focused in understanding the molecular mechanisms underlying the benefits of PGPRs on nitrogen fixation. In this sense, genetic engineering, a powerful tool that has still been poorly exploited in this area, should lead to the generation of strains better adapted to field conditions and with enhanced abilities to help legume–rhizobia symbiosis for effective Cd phytoremediation.

## Conflict of Interest Statement

The authors declare that the research was conducted in the absence of any commercial or financial relationships that could be construed as a potential conflict of interest.
